# Validation of the burnout assessment tool-core symptoms in Spanish veterinarians, sex invariance, and cutoff points

**DOI:** 10.3389/fvets.2024.1454775

**Published:** 2024-10-15

**Authors:** Amparo Osca, Jesús Barrado, Lorena Millán

**Affiliations:** Department of Social and Organizational Psychology, Faculty of Psychology, National University of Distance Education, Madrid, Spain

**Keywords:** Burnout Assessment Tool (BAT-C), invariance analysis, ROC analysis, veterinarians, sex

## Abstract

Notably, most studies on burnout in Veterinary Medicine have used the Maslach Burnout Inventory; however, it has limitations and does not evaluate severe burnout. Therefore, in this study, we validated the Burnout Assessment Tool-Core Symptoms (BAT-C) in a sample of Spanish veterinarians. Its invariance concerning sex and cutoff points were also calculated using the receiver operating characteristic curve analysis and Youden’s index. The sample included 1,215 Spanish veterinarians (70% women). The analyses confirmed that the BAT-C evaluates four dimensions: exhaustion (eight items), mental distance (five items), emotional impairment (five items), and cognitive impairment (five items). Reliability analyses offered adequate results, and the high correlation of the BAT-C with a measure of work stress demonstrates its criterion validity. The invariance analyses showed that it evaluates psychometric guarantees, regardless of the sex of the veterinarian. Regarding the cutoff points, there were more women than men in the high (36.9% vs. 25.5%) and medium (22.4% vs. 18.6%) burnout groups and fewer women in the low burnout group (40.7% vs. 55.9%). Our results support the Spanish version of the BAT-C as a valid instrument to assess the core symptoms of severe burnout among veterinarians. Based on our findings, we provide some practical recommendations to reduce burnout in veterinarians.

## 1 Introduction

Burnout is a mental health problem with high prevalence among veterinary medicine professionals. The burnout levels of veterinarians are higher than those of the general population (1) and are estimated to be approximately 40% higher than those of human doctors (2). The VetsSurvey 2021 report detected high burnout scores worldwide, especially in Argentina, where nearly one in three (31%) veterinary professionals faced ‘completely’ burnout, followed by Spain and the United States of America (19% both) (3). The countries reporting the highest levels of stress are Portugal (87%) and Argentina (79%) although in Spain the percentage is 64%, the same as the world average.

Burnout is a result of chronic work stress and has negative consequences (4, 5). Veterinarians experiencing burnout present with physical (6) and psychological health (7), particularly depression (7). They are also more dissatisfied with their jobs and are more likely to resign (8). These lead to enormous economic losses (9), approximately $1–2 billion annually in the USA (10). However, despite the importance of burnout and the research it has generated, doubts persist regarding its conceptualization and the evaluation instruments used in its measurement. Burnout has been recognized as an occupational phenomenon (11) caused by chronic work stress and not a medical condition; therefore, measures that allow an adequate evaluation are required.

A systematic review of longitudinal studies conducted between 1990 and 2018 supports the notion that continuous exposure to diverse job demands leads to burnout (12). Burnout has been identified in professionals with high social interactions, such as those dedicated to health, as confirmed by different meta-analyses (13–15). Specifically in veterinary medicine, different sources of stress lead to burnout (16–18). The most common stressors are workload, responsibilities, work–family conflicts, tensions with colleagues, financial problems, emotional demands, conflicts with clients, and feelings of danger. Nevertheless, a review suggests analyzing the influence of other moderating variables, such as sex or age, because the prevalence seems higher among women and younger people (5).

Following Maslach and Jackson’s research (19) the evaluation of burnout has focused on measuring three dimensions: emotional exhaustion, depersonalization/cynicism, and loss of personal efficacy. However, some authors have expanded this to include other physical and psychological symptoms. Steffey et al.’s review (9) included physical signs, such as fatigue and somatization, with the eventual development of secondary chronic medical conditions. Burnout was found to accompany high blood pressure and other chronic somatic diseases after adjusting for the effect of age, sex, educational level, or depressive symptoms (20). Mental or neurophysiological symptoms were also observed since people who suffer from burnout react less to physical stimuli (21).

This background has raised the need for clinical burnout indicators and measures that distinguish between employees experiencing burnout and those who are not. In addition, the World Health Organization’s recognition of burnout as an occupational phenomenon caused by chronic work-related stress, but not as a medical condition, reinforces the need for appropriate assessment tools to assess the severity of this problem.

The Maslach Burnout Inventory (MBI) (22) is the most used questionnaire for evaluating burnout. MBI has been used in approximately 88% of all publications (23). Furthermore, it is the questionnaire typically used in Veterinary Medicine (6), although occasionally, other questionnaires, such as the ProQOL, which assess burnout, compassion fatigue, and post-traumatic stress (24) have been used, along with a general measure, the Mayo Clinic Physician Burnout and Wellbeing Scale (25). However, Schaufeli et al. identified three limitations of MBI: conceptual, methodological, and practical. The first refers to the problems encountered when defining the concept of burnout and its dimensions. The second refers to its psychometric deficiencies, specifically, the low reliability of the depersonalization/cynicism and reduced personal efficacy subscales. The third limitation, although associated with the previous ones, refers to difficulty interpreting scores and offering a diagnosis that helps professionals make better decisions. Diagnosing and proposing interventions is difficult if the dimensions of burnout are unclear and no instruments can provide a global and unequivocal score for diagnosing burnout.

Therefore, Schaufeli et al. (26, 27) developed the Burnout Assessment Tool-Core Symptoms (BAT-C) to overcome these limitations. They combined two approaches, one deductive and the other inductive. They developed a measure of clinical burnout based on the two central dimensions: exhaustion and lack of interest or disengagement in work. This includes two other central symptoms: emotional and cognitive impairment. Emotional impairment is the inability to regulate emotions arising from work and cognitive impairment is the inability to regulate cognitive processes such as memory or attention. Notably, both impairments are caused by a lack of energy, leading to burnout and other unmistakable symptoms. Mental distance is a form of psychological detachment from work, and it also changes in the BAT-C compared to the MBI.

A recent systematic review of the BAT-C concluded that it is the most complete assessment instrument, with sufficient content, structure, construct, criterion validity, and internal consistency (12). There are adaptations of the BAT-C scale to different professions, specifically health professionals, such as doctors (28), physiotherapists (29), or nurses (30, 31). However, studies related to veterinarians, professionals who share the problem of burnout with other health professionals, are lacking.

Furthermore, worldwide surveys have indicated that Spanish veterinarians show very high levels of burnout (3). Notably, some Spanish studies (25) reported average levels of burnout. Others reported that 23% of veterinarians experienced burnout, although a higher prevalence was associated with small animal clinicians (75.3%) and women (66.1%) (32). These data indicate an interest in deeper research on this topic by extending the sample size and assessing severe burnout by incorporating indicators of emotional and cognitive problems.

Based on sex differences, women have been found to generally experience higher levels of burnout. A meta-analysis (33) concluded that women are slightly more emotionally exhausted than men, whereas men are depersonalized to an extent compared with women. In the field of medicine, female physicians have disproportionately higher rates of burnout than male physicians (34), approximately higher by 30–60% (35). However, the prevalence rates range from 7.0 to 75.2%, depending on country-specific factors, applied instruments, and cutoff criteria for burnout symptomatology (36). The same trend has been observed in veterinary medicine, whereby female veterinarians experience more exhaustion than male veterinarians (37). Some studies suggest that this difference between men and women is approximately 2:1 (38). There are several possible reasons for these discrepancies. Female veterinarians report higher levels of stress and more difficulties with clients (39), but also more negative coping styles (40). Pohl et al.’s review (1) highlights that female veterinarians perceive more psychological workload. Despite these differences, we have not found studies on whether questionnaires to assess burnout present the same psychometric characteristics for men and women. Specifically, in feminized professions such is veterinary medicine (41), it is useful to calculate the psychometric properties of the assessment instruments for both sexes because if there are differences, it is necessary to know the reason and whether the instruments measure different things or women and men score differently. It is appropriate to determine sex-based invariance because this is a prerequisite for making meaningful comparisons across groups (42, 43).

Therefore, we aimed to validate the BAT-C for Spanish veterinarians, calculate its sex invariance, and establish cutoff scores. In this study, we focused on: (1) analyzing the internal consistency and reliability of the BAT-C; (2) examining its sex invariance, and (3) exploring cutoff scores to identify high levels of stress using receiver operating characteristic (ROC) curve analysis. Calculating the cutoff points for a questionnaire allows differentiating veterinarians at risk of burnout from those who are not and serves to diagnose and make better clinical decisions for treating veterinarians facing burnout.

## 2 Materials and methods

### 2.1 Procedure and sample

Convenience sampling was used in this study. Volunteers were recruited through a private Facebook group of veterinarians, “Ser Veterinario,” with over 1,500 active members. It requires registration for access. This group is used to consult on doubts and complicated cases and share emotionally difficult situations. A Google questionnaire was created, and access was provided between October 1, 2023, and December 17, 2023. Veterinarians were informed that the requested data would only be used for scientific purposes. The Ethics Committee of National Distance University approved all informed consent procedures and protocols (reference: 26-SISH-PSI-2023). SPSS 29.0.2 was used to analyze some of the psychometric properties of the items and AMOS 29 for SPSS 29 to evaluate the measurement model through confirmatory factor analysis (CFA).

Overall, 1,215 veterinarians were involved in small animal care (71.4%), large animal care (13.9%), intensive production (3.7%), animal health (1.8%), agriculture (1.7%), research (1%), sales (1.1%), administration (2.1%), and other jobs (3.2%) answered the questionnaire. A total of 89.1% of veterinarians were engaged in the practice of medicine. Women accounted for 70.8% of respondents (men = 29.03% and others = 0.17%). Their average age was 42.12 (standard deviation [SD] = 10.44) years, and the average length of service as a working professional was 17.19 (SD = 10.04) years. Overall, 62.2% had a bachelor’s degree, and the rest had an additional degree: a professional accreditation of a Spanish veterinarian association (AVEPA) (3.9%), a postgraduate degree (30.6%), a European Diploma (1.3%), or a doctorate (4%). As for their professional situation, 43.4% of the participants were self-employed, and the rest were contract workers. Only 30.1% stated that they worked 40 h per week; 30.1% worked less than 40 h per week (3.5% less than 20 h per week and 26.6% between 21 and 39 h per week), and the rest worked more than 40 h per week (24.3% between 41 and 50 h per week, and 15.5% worked more than 50 h per week).

### 2.2 Instruments

The Spanish version of BAT-C was used.^1^ Table 1 presents the 23 items of their four subscales: exhaustion (eight items: “At work, I feel mentally exhausted”), mental distance (five items: “I struggle to find any enthusiasm for my work”), cognitive impairment (five items: “At work, I have trouble staying focused”), emotional impairment (five items: “At work, I feel unable to control my emotions”). All items were scored on a five-point Likert scale ranging from 1 (disagree) to 5 (agree). Responses were summed and averaged for each subscale. The univariate skewness and kurtosis values for all variables were satisfactory and within the conventional criteria for normality (−3–3 for skewness and − 7–7 for kurtosis) (44).

**Table 1:** Descriptives of the items and subscales of the BAT-C: means, standard deviations, asymmetry, kurtosis and factor loadings.

Items and subscales	$\bar{X}$	SD	A.	K.	F.L.
Exhaustion	3.21	0.84	0.02	−0.50	
1. I feel mentally exhausted	3.62	0.93	−0.40	−0.16	0.74
2. Everything I do requires a great deal of effort	3.40	0.97	−0.06	−0.49	0.71
3. I find it hard to recover my energy	3.44	1.12	−0.25	−0.83	0.76
4. I feel physically exhausted	3.23	1.09	−0.07	−0.74	0.77
5. When I get up in the morning, I lack the energy to start the new day	3.20	1.21	−0.07	−0.99	0.64
6. I want to be active, but somehow, I am unable to manage	2.44	1.02	0.52	−0.17	0.48
7. When I exert myself, I quickly get tired	2.59	1.11	0.45	−0.51	0.54
8. At the end of the day, I feel mentally exhausted and drained	3.75	1.09	−0.55	−0.57	0.70
Mental distance	2.37	0.91	0.63	−0.09	
9. I struggle to find any enthusiasm for my work	2.80	1.24	0.21	−0.90	0.74
10. At work, I do not think much about what I am doing, and I function on autopilot	2.44	1.13	0.40	−0.71	0.55
11. I feel a strong aversion toward my job	2.15	1.18	0.79	−0.32	0.77
12. I feel indifferent about my job	1.89	1.09	1.14	0.46	0.83
13. I’m cynical about what my work means to others	2.57	1.26	0.32	−0.95	0.51
Cognitive impairment	2.39	0.89	0.59	0.06	
14. At work, I have trouble staying focused	2.38	1.01	0.55	−0.11	0.73
15. At work I struggle to think clearly	2.31	0.99	0.60	−0.08	0.74
16. I’m forgetful and distracted at work	2.58	1.18	0.37	−0.79	0.81
17. When I’m working, I have trouble concentrating	2.44	1.08	0.48	−0.48	0.84
18. I make mistakes in my work because I have my mind on other things	2.26	0.97	0.68	0.12	0.73
Emotional impairment	2.63	0.98	0.38	−0.56	
19. At work, I feel unable to control my emotions	2.40	1.09	0.52	−0.44	0.69
20. I do not recognize myself in the way I react emotionally at work	2.22	1.12	0.68	−0.40	0.71
21. During my work I become irritable when things do not go my way	3.21	1.19	−0.12	−0.94	0.75
22. I get upset or sad at work without knowing why	2.70	1.28	0.18	−1.12	0.65
23. At work I may overreact unintentionally	2.61	1.21	0.33	−0.90	0.79

= Mean; SD = Standard Deviation; A. = Asymmetry; K. = Kurtosis; F.L. = Factor Loadings.

Items offered by authors: https://burnoutassessmenttool.be/wp-content/uploads/2020/08/BAT_Spanish-short.pdf.

The Cronbach’s alphas of the four subscales were preliminarily calculated and showed good reliability: 0.91 for exhaustion, 0.83 for emotional distance, 0.91 for cognitive impairment, 0.89 for emotional impairment, and 0.94 for the total scale (Table 2).

**Table 2:** Correlations, descriptives, and reliabilities.

	X	DT	1	2	3	4	5	6	7	8	9	10	11	12
1. Sex	–	–												
2. Age	42.12	10.44	−0.25**											
3. Working hours	–	–	−0.14**	−0.03										
4. Education level	–	–	0.03	−0.16**	0.09**									
5. Employment situation	–	–	−0.14**	0.40**	0.23**	−0.04								
6. Career type	–	–	0.05	−0.15**	0.07*	−0.01	0.22**							
7. BAT–C	2.72	0.74	0.14**	−0.14**	0.06*	−0.02	−0.08**	−0.01	0.94					
8. Exhaustion	3.21	0.84	0.20**	−0.18**	0.11**	0.04	−0.09**	0.01	0.87**	0.91				
9. Mental distance	2.37	0.91	0.03	−0.10**	−0.03	−0.04	−0.10**	−0.03	0.79**	0.58**	0.83			
10. Cognitive impairment	2.40	0.89	0.08**	−0.08**	0.02	−0.05	−0.06*	−0.07*	0.79**	0.54**	0.55**	0.91		
11. Emotional impairment	2.63	0.98	0.12**	−0.10**	0.06*	−0.03	−0.02	0.04	0.81**	0.59**	0.52**	0.56**	0.89	
12. SOS VetMed	3.56	0.72	0.31**	−0.22**	0.07*	0.03	−0.06*	0.17**	0.54**	0.58**	0.36**	0.30**	0.45**	0.92

**p < 0.01; Response scale 1–5; Reliabilities on the diagonal. Sex: 1 = Men, 2 = Women; Education level: 1 = Bachelor; 2 = More than bachelor. Employment situation: Contracted = 1, Self-employed = 2; Career type: clinical practice = 1; nonclinical practice = 2.

## 3 Results

The descriptive results (Table 1) indicated that the mean scores of the exhaustion ( $\bar{X}$ = 3.21; SD = 0.84) and emotional impairment subscales were above the midpoint ( $\bar{X}$ = 2.63; SD = 0.98). The lowest scores were observed for cognitive impairment ( $\bar{X}$ = 2.39, SD = 0.89) and mental distance ( $\bar{X}$ = 2.37, SD = 0.91). The items with the highest scores were “At the end of the day, I feel mentally exhausted and drained” ( $\bar{X}$ = 3.75; SD = 1.09) and “I feel mentally exhausted” ( $\bar{X}$ = 3.62; SD = 0.93), of the exhaustion subscale. The items with the lowest means were “I feel indifferent about my job” ( $\bar{X}$ = 1.89; SD = 1.09) and “I feel a strong aversion toward my job” ( $\bar{X}$ = 2.15; SD = 1.18) on the mental distance subscale.

The results of the Pearson correlation analysis (Table 2) indicated that the female sex was significantly associated with all BAT-C scores except for the mental distance subscale. Age was negatively and significantly correlated with the four subscales of the BAT-C but academic qualifications (bachelor/superior to bachelor) were not associated with any of the BAT-C scores. Regarding the professional variables, working hours/week was positively and significantly associated with exhaustion and emotional impairment. According to the type of professional practice, veterinarians working as clinicians showed significantly less cognitive impairment than non-clinicians. Finally, employed veterinarians scored significantly higher on the BAT-C than self-employed individuals, except for emotional impairment.

The criterion validity of BAT-C was tested by calculating the correlations (Table 2) between their four subscales and one indicator of distress, the sources of stress for medical veterinarians (SOS-VetMed) (17) (Cronbach alpha: 0.93: example items “Communicating bad news to clients about their pets” and “Performing tasks that require more time than expected”) (Table 3). The correlations with SOS-VetMed were highly significant for all BAT-C subscales; the highest correlations were for the “exhaustion” (correlation = 0.58; *p* < 0.001) and “emotional impairment” (correlation = 0.45; *p* < 0.001). The subscale with weaker correlations was “cognitive impairment,” although it was also highly significant (correlation = 0.30; *p* < 0.001).

**Table 3:** Confirmatory factor analysis for BAT-C.

Model	*χ* ^2^	df	*χ*^2^/df	RMSEA	CFI	90% CI
M1. 4 Dimension	1842.12	224	8.22	0.077	0.910	0.04–0.09
M2. 1 Dimension	6370.29	230	27.70	0.148	0.659	0.145–0.151

We performed an exploratory factor analysis (EFA, principal components analysis with direct varimax rotation) for the 23 items in the questionnaire to test the construct validity of BAT-C. We confirmed four factors (eigenvalues >1 and scree plot) that explained the 67.16% of the variance, and all items matched their original factor, except for items 6 and 7; however, the reliability analysis showed that both items contributed positively to the reliability of their subscale. We performed two confirmatory factor analyses (CFA) to validate this structure. We calculated the *χ*^2^ statistic, the comparative fit index (CFI) (45), and the root-mean-square error of approximation (RMSEA) (46). *χ*^2^ by degrees of freedom values should be below than 3.0, but considering the sample size, the rest of the indicators were more appropriate; CFI index should surpass 0.90 (47), and values should be below 0.08 for the RMSEA (48). We tested two different models to find the best factorial solution: Model 1 consisted of four first-order factors. Model 1 was compared with Model 2, which comprised one factor with 23 items (Table 3). Model 1 showed the best fit: CFI = 0.91; RMSEA = 0.077, 90% confidence interval [0.04, 0.09]. According to Hair et al. (49), all standardized factor loadings should be at least 0.5 and ideally at least 0.7; therefore, the results were deemed adequate (Table 1).

### 3.1 Invariance analysis based on sex

We also aimed to test whether the structure of the BAT-C was invariant, that is, whether it was the same regardless of the sex of the surveyed veterinarians. First, the configural invariance was tested, which indicated whether the factor structure was the same for men and women. Second, metric invariance was calculated to analyze whether the magnitude of the factor loadings was the same for both groups. Third, scalar invariance was calculated to test whether the groups responded similarly to the response scale, and strict invariance was calculated to test whether the items had similar residuals. Two criteria were used (50): the difference in RMSEA (ΔRMSEA) was less than 0.01, and the difference in CFI (ΔCFI) was greater than 0.01. The method of estimation of the maximum likelihood was used in all the tested models.

Table 4 shows adequate goodness of fit indices in the unconstrained model (CFI ≥ 0.90 and RMSEA ≤0.05; ΔRMSEA <0.015 and ΔCFI <0.01), indicating that the veterinarians use the same conceptual framework to respond to the items that make up the scale. The results confirm the invariance of the BAT-C with respect to sex, since the criteria for the four types of invariances tested are met.

**Table 4:** Analysis of invariance by sex.

Sex	*χ*^2^ (df)	*χ*^2^/df	CFI	RMSEA (IC 90%)	Comparison	Δ *χ*^2^	ΔCFI	ΔRMSEA
M1. Configuration invariance	2106.10 (448)	4.70	0.907	0.055 (0.053–0.058)				
M2. Metric invariance	2122.29 (467)	4.54	0.907	0.054 (0.052–0.056)	M2 vs. M1	16.19 (19) *p* = 0.644	0.000	−0.001
M3. Scalar invariance	2314.89 (490)	4.72	0.898	0.055 (0.053–0.058)	M3 vs. M2	192.60 (23) *p* = 0.000	−0.009	0.001
M4. Strict invariance	2374.37 (523)	4.54	0.896	0.054 (0.052–0.056)	M4 vs. M3	59.48 (33) *p* = 0.003	−0.002	−0.001

### 3.2 Cutoff points

The cutoff points were calculated for the BAT-C to differentiate veterinarians with high levels of stress from those who did not. Based on a previous report (27), a gold standard related to burnout, work stress, and, specifically, the SOS-VetMed scores (17) was used. Applying ROC analysis, an optimum cutoff value can be calculated to discriminate burnout cases from no cases, considering both specificity and sensitivity (Table 5). According to Schaufeli et al. (26) no critical values for specificity and sensitivity exist because these depend on using the questionnaire under study. For both cases, the maximum threshold corresponds to the 75th percentile and the minimum threshold corresponds to the 25th percentile. The two cutoff points allow distinguish three groups using the so-called traffic light model: a green non-burnout group, an orange group at risk of burnout, and a red burned-out group.

**Table 5:** Sensitivity and specificity values for the Burnout Assessment Tool (BAT-C).

	Specificity		Sensitivity	
	H.C.O.P	L.C.O.P.	H.C.O.P	L.C.O.P.
BAT-C	0.634	0.662	0.762	0.753
Exhaustion	0.819	0.746	0.593	0.699
Mental distance	0.785	0.648	0.510	0.660
Cognitive impairment	0.443	0.533	0.802	0.684
Emotional impairment	0.646	0.753	0.715	0.575

H.C.O.P., High Cut Off Point; L.C.O.P., Low Cut Off Point.

**Figure 1:**
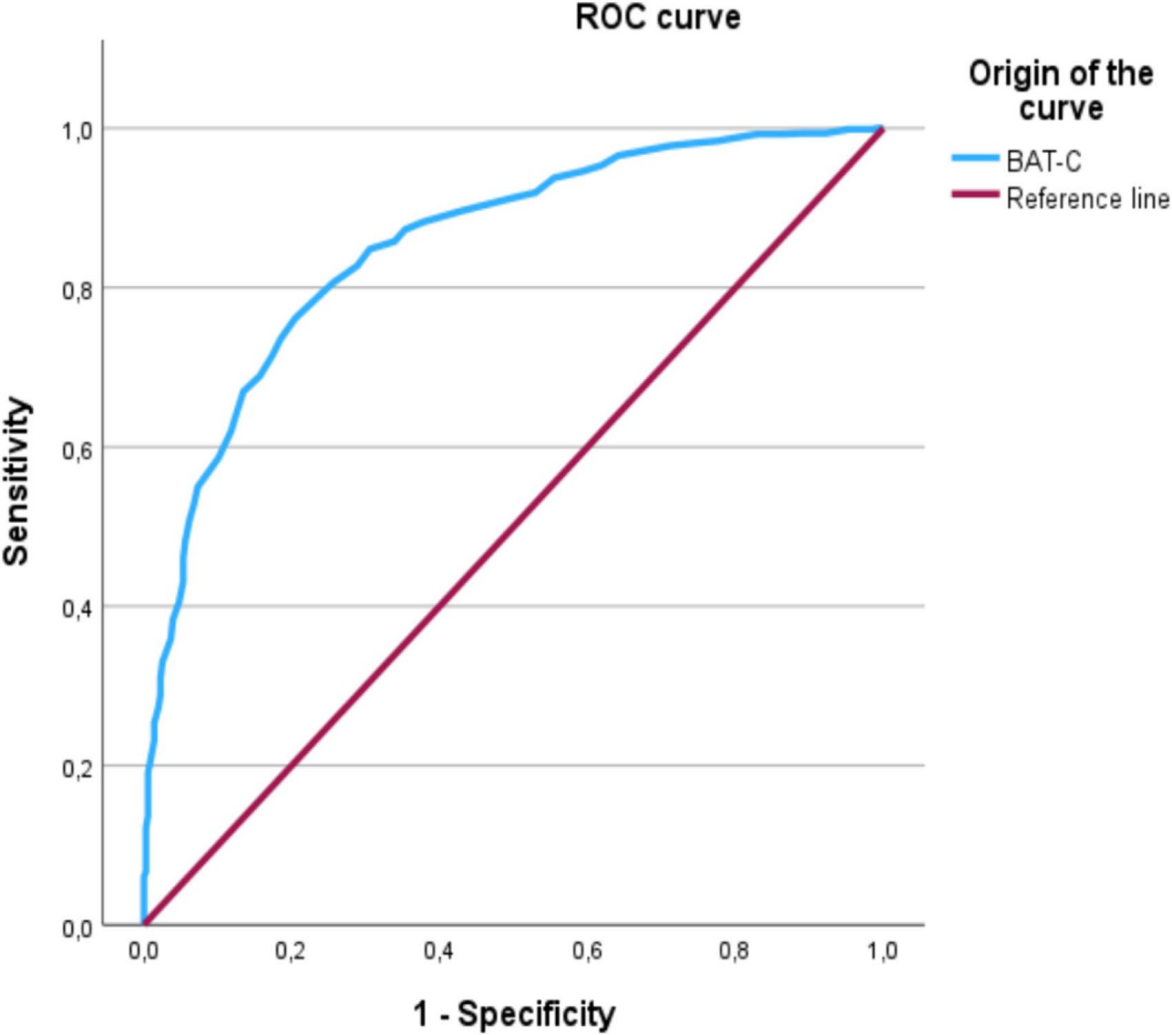
ROC curve for the total score of the BAT-C in the pooled sample. Diagonal segments are produced by ties.

These thresholds enabled us to plot the two ROC curves (one for the upper and lower thresholds), each with a graph of sensitivity (true positive rate) and specificity (false positive rate) for all possible cutoff scores (Figure 1). The area under the curve (AUC) is an indicator of accuracy because it demonstrates the questionnaire’s ability to discriminate between participants experiencing burnout and those who are not.

Finally, the percentages of men and women in the red-, orange-, and green groups were calculated (Table 6). Table 6 shows that there were more women than men in the red (36.9% vs. 25.5%) and orange groups (22.4% vs. 18.6%) and fewer women in the green group (40.7% vs. 55.9%). This trend was maintained in the exhaustion and emotional impairment subscales; however, there were fewer differences in cognitive impairment and differences in mental distance. Notably, more women than men were in the dangerous exhaustion group (red and orange groups, 67.7% vs. 39.0%) and less in the green group (40.7% vs. 55.9%). Similar results were found for emotional impairment, as there were more women than men in the red and orange groups (67.7% vs. 39.0%) and fewer women in the green group (40.7% vs. 55.9%).

**Table 6:** Cutoff values for the BAT-C and its subscales.

Scale	Cutoff traffic light	Scores	*N*	Full scale percentage	*N*	Male percentage	*N*	Female percentage
BAT-C	Red	3.02	396	33.60%	89	25.5%	306	36.9%
	Orange		287	21.3%	80	18.6%	208	22.4%
	Green	2.59	532	45.1%	195	55.9%	337	40.7%
Exhaustion	Red	3.19	608	50.5%	143	39.8%	465	55.1%
	Orange		150	11.6%	38	9.2%	113	12.6%
	Green	2.94	457	37.9%	183	51%	273	32.3%
Mental distance	Red	2.10	676	56.2%	193	53.9%	481	57.1%
	Orange		0	0%	0	0%	0	0%
	Green	2.10	526	43.8%	165	46.1%	361	42.9%
Cognitive impairment	Red	2.90	325	26.9%	81	22.6%	242	28.6%
	Orange		256	20.6%	67	17.2%	191	22%
	Green	2.30	634	52.5%	216	60.2%	418	49.4%
Emotional impairment	Red	2.90	458	38.3%	112	31.7%	346	41.1%
	Orange		356	28.2%	106	26.9%	250	28.6%
	Green	2.10	401	33.5%	146	41.4%	255	30.3%

## 4 Discussion

Notably, many studies have confirmed that burnout is a critical problem for veterinarians; however, most studies have used the MBI, a questionnaire that presents problems in identifying and assessing severe burnout. Therefore, to overcome these limitations, Schaufeli et al. (26) designed the BAT-C, which considers four dimensions: exhaustion, emotional distance, emotional impairment, and cognitive impairment. In this study, we aimed to validate the BAT-C using a sample of Spanish veterinarians. This questionnaire has been validated for many medical professionals; however, it has not been validated for veterinary medicine practitioners or the general Spanish population. Moreover, since it is a feminized profession (1, 41) the psychometric properties of the BAT-C for men and women and the cutoff points allow the differentiation of those who experience burnout from those who do not.

The factorial analyses confirmed the structure of the four subscales, and the reliability analyses showed adequate values, such as those provided by the authors of the set of scales conducted in several countries (51).

The veterinarians surveyed showed medium levels of severe burnout, although they had higher scores in exhaustion, which is consistent with other studies that used this questionnaire among health professionals (29). Scores on the three other subscales are around the midpoint of the midpoint of the measurement scale.

Consistent with other studies that did not use the BAT-C, female veterinarians scored higher on burnout than male veterinarians in Spain (25, 32). However, other variables may also influence these observations since the women in the study were younger than their male colleagues and were contracted workers. These results coincide with findings showing that burnout is negatively associated with the veterinarian’s age (52). Regarding their employment situation, long working hours (53) and working as a clinical veterinarian (54) posed a higher risk of burnout. However, although contracted veterinarians show, in general, more burnout than those who are self-employed, there was no difference in emotional impairment, i.e., both were emotionally disturbed by their work.

The correlations of the BAT-C with external and theoretically related criteria, such as job stress, demonstrate its criterion validity. Specifically, the highest correlations were found mainly with the exhaustion and emotional impairment subscales, indicating that stress primarily affects these dimensions. The weakest correlation was observed for cognitive impairment.

Our second and third objectives were to analyze sex invariance and calculate the cutoff points. Women have been shown to experience more burnout problems; however, none of the reviewed studies tested whether the questionnaires had the same psychometric characteristics regardless of the sex of the veterinarians.

Invariance analysis confirmed that the BAT-C can be used for both men and women (51, 55). Calculations to obtain the cutoff points using an external criterion allowed the sample to be classified into three groups. Using the simile of a traffic light, we identified three levels of burnout: green for veterinarians without burnout, orange for veterinarians at risk of suffering burnout, and red for veterinarians experiencing burnout. Having cutoff points to identify employees at high or medium risk is important for an appropriate diagnosis. The cutoff points found in this study are similar to or slightly lower than, those given by the authors of the questionnaire (56). This may be attributed to the different reference points used in both studies. In the original validation, scores from individuals with a clinical diagnosis of burnout were used, and we considered the job stress scores of the veterinarians themselves. However, the cutoff points are similar in BAT-C score and in emotional impairment subscale, in the emotional exhaustion and in cognitive impairment subscales the differences can be considered small. The mental distance subscale shows higher differences. The veterinarians surveyed show very low scores in mental distance and a score higher than 2.10 is already a red-light indicator.

As expected, there were more women in the red and orange burnout groups and fewer women in the green burnout group than men. These differences were maintained in the exhaustion and emotional impairment, and there were fewer differences in cognitive impairment and hardly any differences in mental distance. We believe this is interesting and novel information, as we have not found any similar study in the field of Veterinary Medicine, either with the BAT-C or any other burnout assessment instruments.

### 4.1 Limitations

This study has some limitations. First, although over 1,200 veterinarians were surveyed, the sample may not be representative of all Spanish veterinarians, but we believe that its size and diversity, and professional experience support our results. In other studies, with smaller samples, the possibility of generalizability of the results is lower. Second, we only used the biological sex variable, and it would also be interesting to evaluate gender identity. Sex and gender are different concepts (57). Practically, the entire sample identified themselves as male or female. However, it would be necessary to investigate whether aspects of gender identity influence burnout in a profession dedicated to health, such as veterinary medicine. It would also be interesting to assess other family (children and partner), and work (workload) variables that may modulate the influence of gender on burnout. Third, all measures were self-reported, which may have increased the risk of common method variance (58); however, in our case, the factorial analysis offered a unidimensional and valid assessment, so such a problem is improbable (59). Nevertheless, future studies, including some objective measures of distress, such as the number of medical visits or medication intake, would be interesting. We did not find any study that analyzed the evolution of burnout in veterinarians over time, so it would be interesting to advance the topic by carrying out longitudinal studies (60). Finally, it would also be interesting to calculate the cutoff points for other variables to determine whether the percentages in each risk group differ (27).

Despite these limitations, this study makes two important contributions to the literature. First, this is the first study to validate the BAT-C and measure severe burnout among Spanish veterinarians. Furthermore, although adaptations of this tool have been made in other health professions, we have not found any that offers information on sex invariance. These results are especially relevant because we have confirmed that possible biases of the questionnaire did not influence the higher scores obtained by women.

Our findings have theoretical and applied connotations. Theoretically, it uses a conceptualization of severe burnout that allows it to be better defined and overcome some of the problems pointed out in this regard. It presents the psychometric characteristics of the BAT-C to evaluate severe burnout in Spanish veterinarians, regardless of their sex. Additionally, BAT-C has several advantages. First, it includes measures of cognitive and emotional impairment. Second, it offers a global measure of burnout that facilitates diagnosis and helps prevent occupational health problems. Third, the subscales allow for a more specific evaluation of four different symptoms and help design more tailored and effective interventions. For example, programs to relieve emotional exhaustion differ from those that seek to reduce the symptoms of cognitive impairment. So, from an applied point of view, reducing exhaustion will require controlling work overload and working hours (61), but reducing cognitive problems require coping cognitive strategies (62). It is also necessary to control working hours; as shown, a high percentage of the veterinarians surveyed work more hours than those regulated by law. Moreover, employers should create safe environments where employees feel comfortable seeking help and foster healthy work cultures (2) especially for women, who are the majority in this profession, and for younger veterinarians. To reduce the perception of burnout, veterinarians should be trained in organizational skills and task design because appropriate and balanced distribution of tasks can reduce physical fatigue and emotional exhaustion (63). These interventions can indirectly contribute to reducing other stressors for veterinarians, such as work–family conflicts and conflicts with colleagues or with animal owners. Finally, by analyzing the cutoff points of this questionnaire, it is possible to diagnose veterinarians who require individual psychological care by specialists. BAT-C can be used in organizational surveys to identify employees at risk of burnout and in clinical treatment settings to identify those experiencing severe burnout, considering the tentativeness of the established limitations.

## 5 Conclusion

This study confirms the use of the BAT-C in diagnosing severe burnout among veterinary medicine professionals in Spain using psychometric guarantees. We also confirmed that it allows us to evaluate, regardless of the sex of the veterinarians, something fundamental in a professional field where the majority are women. As expected, men and women showed different burnout indicators, although women had higher levels of burnout, especially in exhaustion and emotional impairment. Our results show that Spanish veterinarians have burnout scores like those obtained in other studies. Nevertheless, some characteristics make them especially vulnerable, such as age or working long hours as an employee in clinical medicine. This information can help design prevention campaigns to reduce the risks associated with this profession and thus improve the quality of life of Spanish veterinarians.

## Data Availability

The original contributions presented in the study are included in the article/supplementary material, further inquiries can be directed to the corresponding author.

## Footnotes

^1^https://burnoutassessmenttool.be/wp-content/uploads/2020/08/BAT_Spanish-short.pdf
